# Liver stiffness measured by acoustic radiation force impulse elastography predicted prognoses of hepatocellular carcinoma after radiofrequency ablation

**DOI:** 10.1038/s41598-020-58988-3

**Published:** 2020-02-06

**Authors:** Pei-Chang Lee, Yi-You Chiou, Nai-Chi Chiu, Ping-Hsien Chen, Chien-An Liu, Wei-Yu Kao, Teh-Ia Huo, Yi-Hsiang Huang, Ming-Chih Hou, Han-Chieh Lin, Jaw-Ching Wu, Chien-Wei Su

**Affiliations:** 1 Institute of Pharmacology, National Yang-Ming University, Taipei, Taiwan; 2 Faculty of Medicine, School of Medicine, National Yang-Ming University, Taipei, Taiwan; 3 Division of Gastroenterology and Hepatology, Department of Medicine, Taipei, Taiwan; 4 Division of Gastrointestinal Radiology, Department of Radiology, Taipei, Taiwan; 5 Endoscopy Center for Diagnosis and Treatment, Taipei Veterans General Hospital, Taipei, Taiwan; 6 Division of Gastroenterology and Hepatology, Department of Internal Medicine, Taipei Medical University Hospital, Taipei, Taiwan; 7 Division of Gastroenterology and Hepatology, Department of Internal Medicine, School of Medicine, Taipei Medical University, Taipei, Taiwan; 8 Graduate Institute of Clinical Medicine, College of Medicine, Taipei Medical University, Taipei, Taiwan; 9 Institute of Clinical Medicine, School of Medicine, National Yang-Ming University, Taipei, Taiwan; 10 Department of Medical Research, Taipei Veterans General Hospital, Taipei, Taiwan

**Keywords:** Hepatocellular carcinoma, Liver fibrosis

## Abstract

The prognostic factors of patients who undergo radiofrequency ablation (RFA) for hepatocellular carcinoma (HCC) is not fully elucidated. We aimed to investigate the role of liver stiffness (LS) and spleen stiffness (SS) measured by acoustic radiation force impulse (ARFI) elastography in determining the prognoses of patients with HCC after RFA. We prospectively enrolled 173 patients with HCC who underwent ARFI elastography for measurement of LS and SS on the same day of RFA. Overall survival (OS), recurrence-free survival (RFS) after adjusting for competing mortality, and presence of hepatic decompensation were investigated. Patients with LS > 1.5 m/s had significantly shorter OS and RFS than their counterparts. Anti-viral treatment (hazard ratio [HR]: 0.396, p = 0.015) and LS > 1.5 m/s (HR 4.105, p = 0.028) correlated with OS by a multivariate analysis. Besides, serum alpha fetoprotein >10 ng/mL and LS > 1.5 m/s independently predicted poorer RFS. On the other hand, anti-viral treatment (HR: 0.315, p = 0.010), creatinine > 1.5 mg/dL (HR: 9.447, p = 0.006), and SS > 2.7 m/s (HR: 2.869, p = 0.044) predicted a higher risk of hepatic decompensation. In conclusion, LS but not SS measured by ARFI elastography predicted tumor recurrence and OS in RFA-treated HCC; whereas, SS predicted development of hepatic decompensation in these patients.

## Introduction

Hepatocellular carcinoma (HCC) is one of the most common cancers worldwide and contributes to enormous cancer-related deaths annually^1^. Thanks to the well-developed principles of surveillance for HCC in high-risk patients, a large number of patients are diagnosed in the early stage and can be treated by loco-regional ablative therapies, such as radiofrequency ablation (RFA)^2,3^. Nevertheless, the recurrence rates of HCC after RFA are significantly higher than those of surgical resection, in spite of the comparable survival benefit and less serious adverse effects^4–6^. This affects the long-term prognosis of these patients^7^. Besides tumor characteristics, field factors such as the stage of liver fibrosis and the degree of portal hypertension are important risk factors for developing HCC recurrence after treatment^8–10^.

Acoustic radiation force impulse (ARFI) elastography is a reliable tool for assessing the degree of liver stiffness (LS) and predicting the complications of patients with liver cirrhosis^11^. By localizing the area of interest in the ultrasound field, ARFI elastography can be more precisely applied in patients with ascites, liver tumors, and severe obesity^11–13^. It has been declared a higher rate of reproducible measurements and similar predictive value to transient elastography (TE) for significant fibrosis and cirrhosis^14,15^. Moreover, it has been suggested that is accurate and particularly suitable for evaluating advanced liver fibrosis in patients with chronic viral hepatitis^16^.

Several studies have documented the ability to use LS measured by TE in predicting HCC recurrence after local ablation therapy^17,18^. However, evidence of ARFI elastography in this regard is scarce^12^. On the other hand, there is limited data investigated the correlation between spleen stiffness (SS) and the outcomes of HCC^10^, although it has been identified as a predictor to cirrhotic complications, including HCC development and mortality^19–22^. Furthermore, no study to date has investigated SS measured by ARFI elastography in this application. Therefore, this study aimed to evaluate the role of LS and SS measured by ARFI elastography in the prediction of recurrence, overall survival (OS) and hepatic decompensation in patients with HCC after RFA.

## Results

### Demographic characteristics of the patients

As shown in Table 1, the patients in the cohort were predominantly male with a mean age of 69.1 years. Chronic hepatitis B was the most common underlying liver disease, followed by chronic hepatitis C and alcoholic liver disease. All the patients were within Child-Turcotte-Pugh (CTP) grade A at enrollment, but about half of them were classified as the albumin-bilirubin (ALBI) grade 2/3. 27 (15.6%) patients had trace or small esophageal varices, but no one had gastric varices. The median ARFI velocity value for LS was 2.06 m/s (interquartile range IQR, 1.40–3.03), and the median ARFI velocity value for SS was 3.08 m/s (IQR, 2.57–3.51). The cutoff value for LS that provided greatest specificity and sensitivity for predicting mortality was 1.5 m/s, with AUROC of 0.63 (95% CI 0.56–0.70; p = 0.014). Patients with ARFI velocity value > 1.5 m/s for LS had a higher risk of mortality (HR 4.756; 95% CI 1.462–15.467; p = 0.010).

**Table 1:** Baseline characteristics of the study cohort.

Characteristics	Whole cohort	Characteristics	Whole cohort
	(n = 173)		(n = 173)
Age, y	69.1 ± 11.6	ALBI grade 1/2, 3	78/90 (45.1/52.0%)
Sex (male)	107 (61.8%)	Platelet count, K/mm^3^	122 (80–167)
BMI kg/m^2^	25.3 (22.9–28.1)	Albumin, mg/dL	3.8 (3.4–4.2)
HBsAg (+)	81 (46.8%)	Creatinine, mg/dL	0.96 (0.78–1.16)
Anti-HCV (+)	57 (32.9%)	Prothrombin time, INR	1.08 (1.03–1.16)
Anti-viral treatment	87 (50.3%)	ALT, U/L	34 (22–52)
Alcoholism	13 (7.5%)	AST, U/L	37 (26–59)
Tumor size, cm	2.1 ± 0.8	Total bilirubin, mg/dL	0.63 (0.44–1.09)
Tumor number (1/>1)	150/23 (86.7/13.3%)	AAR	1.11 (0.89–1.40)
AFP, ng/mL	9.70 (3.97–60.73)	APRI	0.77 (0.42–1.59)
WBC, /mm^3^	5100 (4000–6250)	Esophageal varices	27 (15.6%)
NLR	2.11 (1.51–2.88)	ARFI, m/s (LS)	2.06 (1.40–3.03)
CRP, mg/dL	0.53 (0.15–1.65)	ARFI, m/s (SS)	3.08 (2.57–3.51)

AAR, AST to ALT ratio; AFP, alpha fetoprotein; ALBI grade, albumin-bilirubin grade; ALT, alanine aminotransferase; AST, aspartate aminotransferase; APRI, AST to platelet ratio index; ARFI, acoustic radiation force impulse; BMI, body mass index; HBsAg, hepatitis B surface antigen; HCV, hepatitis C; INR, international normalized ratio; LS, liver stiffness; NA, not adopted; NLR, neutrophil to lymphocyte ratio; NS, not significant; PALBI grade, platelet-albumin-bilirubin grade; SS, spleen stiffness.

**Figure 1:**
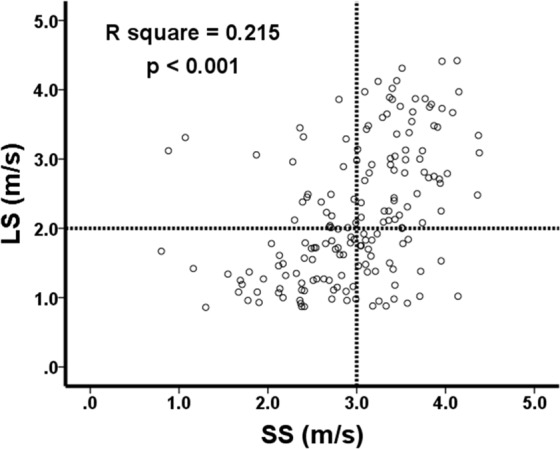
The correlation between LS and SS.

When divided by the optimal cut-off value of 1.5 m/s for LS, 126 patients were classified as having significant liver fibrosis, and the other 47 patients were not. Patients with LS > 1.5 m/s had significantly greater serum levels of alpha-fetoprotein (AFP), prothrombin time, alanine aminotransferase (ALT), aspartate aminotransferase (AST), total bilirubin, blood neutrophil counts as well as greater AST to platelet ratio index (APRI) and ALBI grade, but lower platelet counts and serum albumin level compared with their counterpart (Table 2). However, the rates of viral etiology, anti-viral treatment, and alcoholism were comparable between these two groups of patients. Besides, higher ARFI velocity values for SS were also found in patients with significant LS; and there was a modest positive association between LS and SS measured by ARFI velocity **(**R^2^ = 0.215, p < 0.001, Fig. 1**)**. In addition, more hepatic decompensated events, especially formation of ascites, and death developed in patients with significant LS during the follow-up period.

**Table 2:** Clinical features of patients associated with significant liver stiffness.

Characteristics	Before propensity score matching			After propensity score 1:1 matching^#^		
	Significant LS (LS > 1.5 m/s)	Non-significant LS (LS ≤ 1.5 m/s)	*p* value	Significant LS (LS > 1.5 m/s)	Non-significant LS LS ≤ 1.5 m/s	*p* value
	(n = 126)	(n = 47)		(n = 47)	(n = 47)	
Age, y	68.8 ± 10.9	69.8 ± 13.6	0.675	68.9 ± 11.9	69.8 ± 13.6	0.800
Sex (male)	78 (61.9%)	29 (61.7%)	0.981	32 (68.1%)	29 (61.7%)	0.517
BMI kg/m^2^	25.5 (22.9–28.5)	24.8 (21.7–27.7)	0.177	26.5 (22.9–28.9)	24.8 (21.7–27.7)	0.099
HBsAg (+)	57 (45.2%)	24 (51.1%)	0.495	22 (46.8%)	24 (51.1%)	0.680
Anti-HCV (+)	45 (35.7%)	12 (25.5%)	0.205	13 (27.7%)	12 (25.5%)	0.815
Anti-viral treatment	65 (51.6%)	22 (46.8%)	0.576	26 (55.3%)	22 (46.8%)	0.409
Alcoholism	11 (8.7%)	2 (4.3%)	0.321	5 (10.6%)	2 (4.3%)	0.239
Tumor size, cm	2.2 ± 0.8	2.1 ± 0.7	0.620	2.2 ± 08	2.1 ± 0.7	0.578
Tumor number (1/>1)	112/14 (88.9/11.1%)	38/9 (80.9/19.1%)	0.358	42/5 (89.4/10.6%)	38/9 (80.9/19.1%)	0.509
AFP, ng/mL	11.96 (5.07–69.94)	4.69 (2.63–20.32)	0.012	10.70 (4.95–26.11)	8.69 (4.63–20.32)	0.068
ALBI grade 1/2, 3	47/76 (37.3/60.3%)	31/14 (66.0/29.8%)	0.001	22/23 (46.8/48.9%)	31/14 (66.0/29.8%)	0.061
PALBI grade 1/2, 3	65/58 (51.6/46.0%)	30/15 (63.8/31.9%)	0.265	31/14 (66.0/29.8%)	30/15 (63.8/31.9%)	0.971
WBC, /mm^3^	5600 (4600–7100)	4700 (3700–5900)	0.001	5600 (4600–7100)	5400 (4100–6000)	0.036
NLR	2.03 (1.45–2.74)	2.41 (1.72–3.17)	0.174	2.22 (1.53–2.93)	2.41 (1.2–3.17)	0.335
Platelet count, K/mm^3^	104 (72–139)	172 (133–209)	<0.001	114 (85–145)	172 (133–209)	<0.001
Albumin, mg/dL	3.7 (3.3–4.1)	4.1 (3.8–4.4)	<0.001	3.7 (3.4–4.2)	4.1 (3.8–4.4)	0.003
Creatinine, mg/dL	0.99 (0.77–1.16)	0.95 (0.82–1.16)	0.886	1.06 (0.81–1.30)	0.95 (0.82–1.16)	0.149
Prothrombin time, INR	1.11 (1.05–1.18)	1/03 (1.00–1.08)	<0.001	1.08 (1.04–1.13)	1.03 (1.00–1.08)	<0.001
ALT, U/L	37 (26–61)	25 (18–33)	<0.001	36 (23–48)	25 (18–33)	0.003
AST, U/L	45 (31–68)	27 (20–37)	<0.001	38 (26–59)	27 (20–37)	0.001
Total bilirubin, mg/dL	0.75 (0.48–1.19)	0.56 (0.39–0.81)	0.007	0.56 (0.39–0.81)	0.56 (0.39–0.80)	0.976
AAR	1.12 (0.91–1.41)	1.07 (0.84–1.39)	0.338	1.11 (0.89–1.44)	1.07 (0.84–1.39)	0.790
APRI	0.97 (0.57–2.03)	0.34 (0.22–0.56)	<0.001	0.81 (0.49–1.37)	0.34 (0.22–0.56)	<0.001
CRP, mg/dL	0.74 (0.15–3.26)	0.38 (0.13–1.13)	0.173	0.79 (0.11–3.50)	0.38 (0.13–1.13)	0.353
ARFI, m/s (LS)	2.49 (1.95–3.30)	1.17 (1.02–1.31)	<0.001	2.28 (1.91–3.13)	1.17 (1.02–1.31)	<0.001
ARFI, m/s (SS)	3.24 (2.78–3.59)	2.54 (2.14–3.02)	<0.001	3.09 (2.65–3.54)	2.54 (2.14–3.02)	<0.001
Follow-up events						
Hepatic decompensation*	48 (38.1%)	8 (17.0%)	0.008	11 (23.4%)	8 (17.0%)	0.441
Ascites formation	47 (37.3%)	8 (17.0%)	0.011	11 (23.4%)	8 (17.0%)	0.441
Variceal bleeding	5 (4.0%)	2 (4.3%)	0.932	1 (2.1%)	2 (4.3%)	0.557
Hepatic encephalopathy	13 (10.3%)	4 (8.5%)	0.722	2 (4.3%)	4 (8.5%)	0.399
Death	35 (27.8%)	3 (6.4%)	0.002	10 (21.3%)	3 (6.4%)	0.036

^#^Propensity score matching for tumor size, tumor number, serum level of AFP, total bilirubin, status of chronic viral hepatitis.

^*^Hepatic decompensation newly developed in the follow-up period.

AAR, AST to ALT ratio; AFP, alpha fetoprotein; ALBI grade, albumin-bilirubin grade; ALT, alanine aminotransferase; AST, aspartate aminotransferase; APRI, AST to platelet ratio index; ARFI, acoustic radiation force impulse; BMI, body mass index; HBsAg, hepatitis B surface antigen; HCV, hepatitis C; INR, international normalized ratio; LS, liver stiffness; NA, not adopted; NLR, neutrophil to lymphocyte ratio; NS, not significant; PALBI grade, platelet-albumin-bilirubin grade; SS, spleen stiffness.

After propensity score 1:1 matched by number of tumors, viral hepatitis status, serum levels of AFP and total bilirubin which would have impacts on adverse tumor events, patients with significant LS still had greater levels of prothrombin time, ALT,AST, APRI, white blood cell counts, but lower serum albumin and platelet counts. Hepatic decompensated events did not develop more in patients with significant LS; but more patients died in this group.

### OS in patients with HCC post-RFA

**Figure 2:**
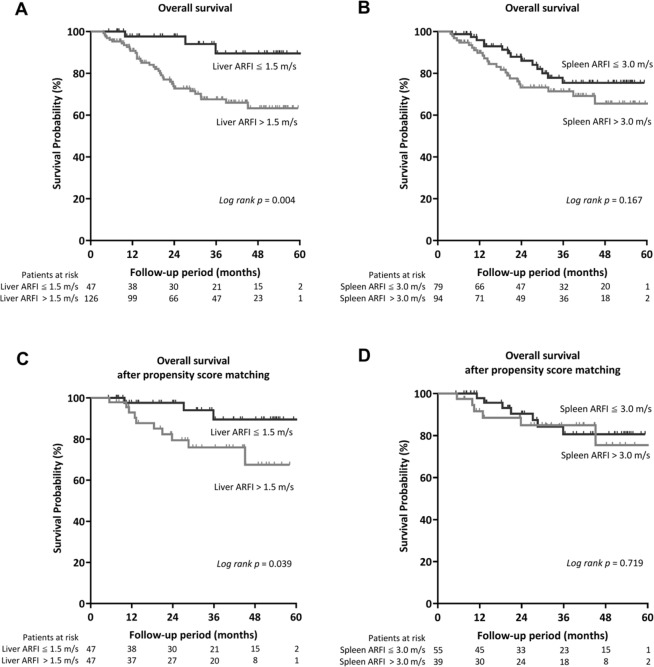
Comparison of the OS of patients with HCC after RFA stratified by (**A**) LS and (**B**) SS and after matching by propensity score (**C**,**D**).

During the median follow-up period of 27.7 months (IQR, 13.3–44.6), 38 (22.0%) patient deaths occurred. No patients underwent liver transplantation during the follow-up period. The cumulative 1, 2, and 5-year OS rates were 93.8%, 80.3%, and 71.4%, respectively. Stratified by the status of LS, the cumulative 1, 2, and 5-year OS rates were 97.6%, 94.0%, and 89.5% in patients with ARFI ≤ 1.5 m/s, while they were 92.3%, 74.4%, and 65.0% in those with ARFI > 1.5 m/s, respectively (*p* = 0.004, Fig. 2A). On the other hand, the best cutoff value for SS was 3.0 m/s, with AUROC of 0.61 (95% CI 0.53–0.68; p = 0.047). However, the OS rates were not significantly different when stratified by SS status (*p* = 0.167, Fig. 2B). After propensity score matching, a significant better OS was still observed in patients with ARFI ≤ 1.5 m/s for LS. Nevertheless, no significant difference of OS could be divided by the status of SS (Fig. 2C,D).

By the multivariate analysis which included dimensional ARFI velocity value for LS, anti-viral treatment was the only predictor of survival benefit (model 1). After including dichotomous value of LS into analysis (model 2), both anti-viral treatment (hazard ratio [HR]: 0.396, *p* = 0.015) and LS > 1.5 m/s (HR: 4.105, *p* = 0.028) were independent predictors to OS in patients with HCC after RFA (Table 3**)**. However, SS was not significantly associated with OS even among patients with esophageal varices or thrombocytopenia < 100 K/cumm at baseline (Supplementary Table).

**Table 3:** Analysis of factors associated with OS.

		Univariate			Multivariate (Model I)			Multivariate (Model II)		
		HR	95% CI	*p* value	HR	95% CI	*p* value	HR	95% CI	*p* value
Age, y	>60 vs. ≦60	2.674	0.949–7.537	0.063			NA			NA
Sex	Male vs. Female	0.574	0.304–1.087	0.088			NA			NA
BMI kg/m^2^	>25.0 vs. ≦25.0	1.065	0.549–2.067	0.853			NA			NA
HBsAg (+)	Yes vs. No	0.400	0.198–0.806	0.010			NS			NS
Anti-HCV (+)	Yes vs. No	1.298	0.677–2.488	0.432			NA			NA
Antiviral treatment	Yes vs. No	0.444	0.229–0.861	0.016	0.415	0.198–0.872	0.020	0.396	0.188–0.833	0.015
Alcoholism	Yes vs. No	1.483	0.526–4.183	0.456			NA			NA
Tumor size, cm	>2 vs. ≦2	1.565	0.822–2.981	0.173			NA			NA
Tumor number	>1 vs. 1	1.465	0.645–3.328	0.361			NA			NA
AFP, ng/mL	>10 vs. ≦10	1.140	0.593–2.191	0.695			NA			NA
ALBI grade	Grade 2 vs. 1	2.141	1.014–4.522	0.046			NS			NS
	Grade 3 vs. 1	5.153	1.754–15.138	0.003			NS			NS
Platelet count	≦100 K vs. >100 K	2.111	1.113–4.002	0.022			NS			NS
Albumin, mg/dL	≦3.5 vs. >3.5	2.538	1.331–4.839	0.005			NA			NA
Creatinine, mg/dL	>1.5 vs. ≦1.5	2.347	0.981–5.619	0.055			NA			NA
Prothrombin time, INR	>1.2 vs. ≦1.2	2.170	1.051–4.477	0.036			NS			NS
ALT, U/L	>40 vs. ≦40	1.662	0.880–3.140	0.118			NA			NA
AST, U/L	>45 vs. ≦45	2.519	1.306–4.859	0.006			NS			NS
Total bilirubin, mg/dL	>2.0 vs. ≦2.0	2.058	0.903–4.687	0.086			NA			NA
AAR	>1.0 vs. ≦1.0	2.759	1.211–6.286	0.016			NA			NA
APRI	>1.0 vs. ≦1.0	2.582	1.339–4.980	0.005			NA			NA
NLR	>2.0 vs. ≦2.0	1.258	0.656–2.410	0.490			NA			NA
ARFI, m/s (liver)		1.547	1.114–2.147	0.009			NS			NA
	>1.5 vs. ≦1.5	4.756	1.462–15.467	0.010			NA	4.105	1.160–14.524	0.028
ARFI, m/s (spleen)		1.601	0.746–3.103	0.134			NA			NA
	>3.0 vs. ≦3.0	1.584	0.820–3.064	0.171			NA			NA

AAR, AST to ALT ratio; AFP, alpha fetoprotein; ALBI grade, albumin-bilirubin grade; ALT, alanine aminotransferase; AST, aspartate aminotransferase; APRI, AST to platelet ratio index; ARFI, acoustic radiation force impulse; BMI, body mass index; CI, confidence interval; HBsAg, hepatitis B surface antigen; HCV, hepatitis C; HR, hazard ratio; INR, international normalized ratio; NA, not adopted; NLR, neutrophil to lymphocyte ratio; NS, not significant; PALBI grade, platelet-albumin-bilirubin grade.

Model 1(2): multivariate analysis with adoption of dimensional (dichotomous) ARFI velocity value of liver stiffness.

### RFS in patients with HCC post RFA

**Figure 3:**
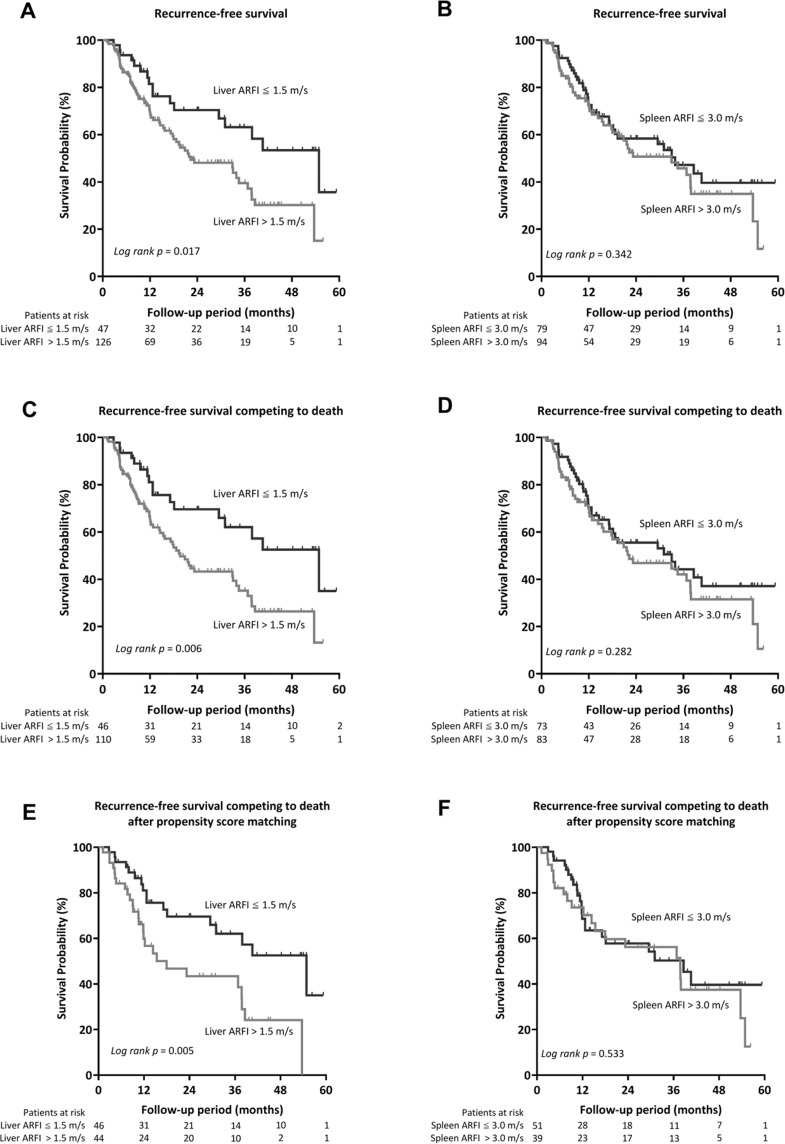
Comparison of the RFS of patients with HCC after RFA stratified by (**A**) LS and (**B**) SS; estimated by competing risk model (**C**,**D**) and also after matching by propensity score (**E**,**F**).

During the follow-up period, 80 (46.2%) patients developed tumor recurrence. The cumulative recurrence-free survival (RFS) rates at 1, 2, and 5 years were 73.2%, 54.1%, and 23.6%, respectively. As shown in Fig. 3A, patients with an ARFI velocity value > 1.5 m/s for LS at baseline had significantly shorter RFS compared to those with LS ≤ 1.5 m/s (22.3 vs. 54.9 months, *p* = 0.017). The cumulative RFS rates at 1, 2, and 5 years were 81.5%, 70.4%, and 35.6% in patients with LS ≤ 1.5 m/s, while they were 70.1%, 47.9%, and 15.0% and in those with ARFI > 1.5 m/s, respectively. However, the RFS rates were comparable according to ARFI values for SS (*p* = 0.342, Fig. 3B).

In the model analyzed competing risk of death, patients with LS > 1.5 m/s still had significantly shorter RFS compared with their counterparts (19.6 vs. 54.9 months, *p* = 0.006). Moreover, significant difference of RFS was also observed according to LS after propensity score matching in the competing risk model (18.0 vs. 54.9 months, *p* = 0.005) (Fig. 3C–F).

By including dimensional LS value into multivariate analysis in the competing risk model, a higher baseline AFP level was the only predictor to RFS after RFA (model 1). After including dichotomous value of LS into analysis (model 2), a higher baseline AFP level (subdistribution hazard ration [SHR]: 1.701, *p* = 0.040) and significant LS > 1.5 m/s (SHR: 2.000, *p* = 0.027) could independently predict HCC recurrence after RFA **(**Table 4**)**. Nevertheless, SS was not significantly associated with RFS even among patients with clinically significant portal hypertension (Supplementary Table).

**Table 4:** Competing risk analysis of factors associated with RFS.

		Univariate			Multivariate (Model I)			Multivariate (Model II)		
		SHR	95% CI	*p* value	SHR	95% CI	*p* value	SHR	95% CI	*p* value
Age, y	>60 vs. ≦60	2.150	1.184–3.903	0.012			NS			NS
Sex	Male vs. Female	1.397	0.860–2.268	0.177			NA			NA
BMI kg/m^2^	>25.0 vs. ≦25.0	1.115	0.708–1.758	0.638			NA			NA
HBsAg (+)	Yes vs. No	1.113	0.716–1.730	0.635			NA			NA
Anti-HCV (+)	Yes vs. No	0.924	0.573–1.488	0.744			NA			NA
Antiviral treatment	Yes vs. No	0.835	0.537–1.299	0.425			NA			NA
Alcoholism	Yes vs. No	0.774	0.283–2.119	0.619			NA			NA
Tumor size, cm	>2 vs. ≦2	1.377	0.886–2.140	0.155			NA			NA
Tumor number	>1 vs. 1	1.757	0.999–3.090	0.050			NA			NA
AFP, ng/mL	>10 vs. ≦10	1.794	1.131–2.846	0.013	1.873	1.132–3.098	0.015	1.701	1.025–2.824	0.040
ALBI grade	Grade 2 vs. 1	0.996	0.626–1.583	0.996			NA			NA
	Grade 3 vs. 1	1.614	0.631–4.130	0.318			NA			NA
Platelet count	≦100 K vs. >100 K	1.409	0.902–2.201	0.131			NA			NA
Albumin, mg/dL	≦3.5 vs. >3.5	1.491	0.928–2.394	0.099			NA			NA
Creatinine, mg/dL	>1.5 vs. ≦1.5	2.122	1.016–4.432	0.045			NS			NS
Prothrombin time, INR	>1.2 vs. ≦1.2	1.216	0.670–2.209	0.520			NA			NA
ALT, U/L	>40 vs. ≦40	1.135	0.724–1.780	0.582			NA			NA
AST, U/L	>45 vs. ≦45	1.478	0.930–2.349	0.098			NA			NA
Total bilirubin, mg/dL	>2.0 vs. ≦2.0	1.289	0.643–2.584	0.474			NA			NA
AAR	>1.0 vs. ≦1.0	1.385	0.850–2.257	0.190			NA			NA
APRI	>1.0 vs. ≦1.0	1.329	0.832–2.123	0.234			NA			NA
NLR	>2.0 vs. ≦2.0	0.766	0.491–1.194	0.239			NA			NA
ARFI, m/s (liver)		1.273	1.015–1.596	0.037			NS			NA
	>1.5 vs. ≦1.5	2.102	1.221–3.620	0.007			NA	2.000	1.083–3.693	0.027
ARFI, m/s (spleen)		1.184	0.848–1.653	0.320			NA			NA
	>3.0 vs. ≦3.0	1.275	0.818–1.987	0.283			NA			NA

AAR, AST to ALT ratio; AFP, alpha fetoprotein; ALBI grade, albumin-bilirubin grade; ALT, alanine aminotransferase; AST, aspartate aminotransferase; APRI, AST to platelet ratio index; ARFI, acoustic radiation force impulse; BMI, body mass index; CI, confidence interval; HBsAg, hepatitis B surface antigen; HCV, hepatitis C; INR, international normalized ratio; NA, not adopted; NLR, neutrophil to lymphocyte ratio; NS, not significant; PALBI grade, platelet-albumin-bilirubin grade; SHR, subdistribution hazard ratio.

Model 1(2): multivariate analysis with adoption of dimensional (dichotomous) ARFI velocity value of liver stiffness.

### Development of hepatic decompensation in patients with HCC post RFA

During the follow-up period, 56 patients developed hepatic decompensation, in which 55 patients had ascites, 7 had variceal bleeding, and 17 developed hepatic encephalopathy. The cutoff value for LS to predict hepatic decompensation was 2.0 m/s, with AUROC of 0.65 (95% CI 0.56–0.72; p = 0.003). Patients with LS > 2.0 m/s had a higher risk of decompensation (OR 2.969; 95% CI 1.509–5.841; p = 0.002). Besides, the best cutoff value of SS for hepatic decompensation was 2.7 m/s, with AUROC of 0.66 (95% CI 0.58–0.74; p = 0.001). Higher SS > 2.7 m/s was associated with a significantly higher risk of hepatic decompensation (OR 4.870; 95% CI 2.033–11.663; p < 0.001).

In the multivariate analysis that including dimensional ARFI values of LS and SS (model 1), antiviral treatment, serum levels of creatinine and ALT, and ARFI velocity value for SS were independent predictors to hepatic decompensation. In the model 2 which included dichotomous ARFI values, anti-viral treatment, serum creatinine > 1.5 mg/dL, and SS > 2.7 m/s independently predict hepatic decompensation (Table 5).

**Table 5:** Risk analysis of factors associated with hepatic decompensation.

		Univariate			Multivariate (Model I)			Multivariate (Model II)		
		OR	95% CI	*p* value	OR	95% CI	*p* value	OR	95% CI	*p* value
Age, y	>60 vs. ≦60	2.253	0.911–5.571	0.079			NA			NA
Sex	Male vs. Female	1.073	0.528–2.179	0.846			NA			NA
BMI kg/m^2^	>25.0 vs. ≦25.0	0.913	0.455–1.830	0.797			NA			NA
HBsAg (+)	Yes vs. No	0.462	0.230–0.928	0.030			NS			NS
Anti-HCV (+)	Yes vs. No	1.785	0.870–3.660	0.114			NA			NA
Antiviral treatment	Yes vs. No	0.468	0.234–0.936	0.032	0.320	0.127–0.804	0.015	0.315	0.131–0.758	0.010
Alcoholism	Yes vs. No	3.714	0.997–13.834	0.050			NS			NS
Tumor size, cm	>2 vs. ≦2	1.817	0.913–3.617	0.089			NA			NA
Tumor number	>1 vs. 1	1.343	0.522–3.452	0.541			NA			NA
AFP, ng/mL	>10 vs. ≦10	1.423	0.707–2.866	0.323			NA			NA
ALBI grade	Grade 2 vs. 1	3.159	1.489–6.702	0.003			NS			NS
	Grade 3 vs. 1	4.357	0.970–19.580	0.055			NS			NS
Platelet count	≦100 K vs. >100 K	2.826	1.395–5.723	0.004			NS			NS
Albumin, mg/dL	≦3.5 vs. >3.5	4.073	1.922–8.633	0.001			NA			NA
Creatinine, mg/dL	>1.5 vs. ≦1.5	3.517	1.056–11.716	0.041	9.324	1.442–60.289	0.019	9.447	1.910–46.740	0.006
Prothrombin time, INR	>1.2 vs. ≦1.2	3.631	1.505–8.763	0.004			NS			NS
ALT, U/L	>40 vs. ≦40	3.194	1.576–6.472	0.001	3.935	1.121–13.815	0.033			NS
AST, U/L	>45 vs. ≦45	2.691	1.309–5.533	0.007			NS			NS
Total bilirubin, mg/dL	>2.0 vs. ≦2.0	1.571	0.526–4.694	0.418			NA			NA
AAR	>1.0 vs. ≦1.0	2.267	1.036–4.961	0.041			NA			NA
APRI	>1.0 vs. ≦1.0	3.565	1.712–7.423	0.001			NA			NA
NLR	>2.0 vs. ≦2.0	1.21	0.610–2.420	0.579			NA			NA
ARFI, m/s (liver)		1.663	1.150–2.405	0.007			NS			NA
	>2.0 vs. ≦2.0	2.969	1.509–5.841	0.002			NA			NS
ARFI, m/s (spleen)		2.806	1.504–5.234	0.001	2.664	1.108–6.404	0.029			NA
	>2.7 vs. ≦2.7	4.870	2.033–11.663	<0.001			NA	2.869	1.030–7.993	0.044

AAR, AST to ALT ratio; AFP, alpha fetoprotein; ALBI grade, albumin-bilirubin grade; ALT, alanine aminotransferase; AST, aspartate aminotransferase; APRI, AST to platelet ratio index; ARFI, acoustic radiation force impulse; BMI, body mass index; CI, confidence interval; HBsAg, hepatitis B surface antigen; HCV, hepatitis C; HR, hazard ratio; INR, international normalized ratio; NA, not adopted; NLR, neutrophil to lymphocyte ratio; NS, not significant; PALBI grade, platelet-albumin-bilirubin grade.

Model 1(2): multivariate analysis with adoption of dimensional (dichotomous) ARFI velocity value of liver stiffness and splenic stiffness.

## Discussion

This study investigated the role of LS and SS measured by ARFI velocity in predicting the patients’ outcomes after RFA. It showed that higher LS values measured by ARFI velocity could be a significant predictor of both HCC recurrence and OS in these patients, but no significant role of SS could be identified in the evaluation of post-RFA outcomes. As patients who receive RFA for HCC usually have more advanced chronic liver disease, or more severe portal hypertension than others who undergo surgical resection. By using more reliable tools for stiffness measurement, such as ARFI elastography, our findings could be applied in clinical practice to optimize the follow-up program for patients with higher risks of recurrence or mortality after RFA treatment.

For patients with early-stage HCC, RFA could provide acceptable long-term OS rates that are comparable or only slightly inferior to that of surgical resection, but the recurrence rates after RFA are still high^23,24^. Our previous study showed that the cumulative 10-year OS and RFS rates after RFA were 48.7% and 12.4%, respectively^23^. To improve the outcomes of patients, it is crucial to elucidate the mechanism and identify the risk factors of tumor recurrence after RFA. Identified predictors of HCC recurrence after curative therapies include tumor factors (including tumor size, number, tumor cell differentiation, vascular invasion, extra-hepatic metastasis, and serum AFP level), liver functional reserve (such as serum albumin level, platelet count, and portal hypertension), and field factors in the background liver (including the grade of hepatic inflammation and steatosis and the stage of liver fibrosis)^12,18,25–29^.

As the tumor factors might be less apparent in determining the outcomes of patients with early-stage HCC, field factors may play a more important role in tumor recurrence after curative treatments for such patients. To date, only one study from Korea has proposed that the ARFI velocity value of LS assessed at the time of RFA can independently predict the risk of HCC recurrence after treatment^12^. However, that study recruited a relatively small number of patients (n = 120) and could not find a predictor of survival benefit.

In this study, we enrolled 173 HCC patients and confirmed the ability of ARFI elastography to predict not only tumor recurrence but also OS after RFA. The optimal cut-off value of ARFI velocity to predict HCC recurrence was 1.6 m/s in the Korean population^12^. In our cohort, the optimal cut-off value was 1.5 m/s, which could be used to predict both tumor recurrence and OS after RFA effectively. This corresponds to a previous study, in which the cut-off value of ARFI velocity to predict cirrhosis was 1.52 m/s^30^. Thus, the optimal cut-off value in our study seems reasonable because liver cirrhosis is confirmed as an important risk factor for HCC recurrence and mortality after treatment^8^.

In our cohort, patients with significant liver fibrosis measured by ARFI velocity (>1.5 m/s) had poorer liver functional reserve and lower rates of OS and RFS compared to those with non-significant liver fibrosis. The differences of survival benefit and tumor recurrence were still prominent according to LS even after matching for tumor factors and liver reserve. For such patients, improving the prognosis might require following-up patients more closely, arranging salvage liver transplantation in cases of tumor recurrence or liver decompensation, or prescribing adjuvant therapy (such as molecular target therapy or immune check point inhibitors) after RFA. More prospective studies are needed to validate this concept.

Patients who had non-significant liver fibrosis had 5-year RFS rates of only 35.6%, but the long-term outcome was excellent with a 5-year OS rate of 89.5%. This might be due to the patients undergoing a strict surveillance program to detect recurrence after RFA. They could still undergo curative treatment modalities when HCC recurs due to the well-preserved liver function and early tumor stage. This indicates that liver functional reserve and surveillance programs for tumor recurrence are crucial in determining the long-term outcomes of HCC patients who have undergone RFA.

During the long-term follow-up after RFA for HCC, significantly negative effects on OS were found for age, prothrombin activity, advanced CTP grade, tumor size, tumor number, serum level of AFP, and the presence of porto-systemic collateral vessels^23,24,31–33^. LS measured by two-dimensional shear-wave elastography has been reported as a significant predictive factor for OS after RFA for HCC^29^. However, in other studies in Korea, LS measured by TE or ARFI elastography did not have effective prediction performance for OS in HCC patients who received RFA^12,18^. In our study, we identified that an ARFI velocity cut-off value of 1.5 m/s for LS could significantly predict the OS of patients who underwent RFA for HCC. Moreover, patients with significant liver fibrosis measured by ARFI velocity had a relatively poorer liver function as well as higher aminotransferases level and blood neutrophil counts compared to their counterparts. These findings suggested that LS may not only indicate hepatic fibrosis but also correlated with hepatic and systemic inflammation that were closely associated with tumor outcomes^34,35^. On the other hand, antiviral therapy was associated with a lower risk of mortality after RFA, which is consistent with previous studies reporting that ongoing viral replication and antiviral therapy could be used to determine the prognoses of patients with early-stage HCC who underwent RFA^36,37^.

SS has been investigated as a non-invasive marker of portal hypertension. It has a close correlation with the hepatic venous pressure gradient and has been identified as a predictor of cirrhotic complications^19–22^. According to a previous study, cirrhotic patients with an ARFI velocity value for SS less than 3.25 m/s had a 98.8% probability of not developing hepatic decompensation, and patients with a value greater than 3.43 m/s had a 75.8% probability of mortality^38^. On the other hand, SS measured by TE was suggested to be a predictor of late HCC recurrence at 24 months after liver resection in a recent Italian study^10^. However, the role of SS measured by ARFI velocity in determining the outcomes of HCC after RFA has not been well investigated before. In this study, we identified that SS > 2.7 m/s was associated with a significantly higher risk of hepatic decompensation in HCC patients after RFA treatment even with good liver reserves (Child-Pugh A). However, the status of SS failed to predict survival benefit or tumor recurrence after RFA treatment for HCC even in patients with clinically significant portal hypertension.

To the best of our knowledge, this study is the largest one to compare LS and SS measured by ARFI in determining the prognosis of HCC patients after RFA. Although there was a modest positive correlation between LS and SS measured by ARFI velocity, we could not identify a role of SS in predicting the risk of recurrence or mortality after RFA, even when judged by different cut-off values with increments of 0.1 from 3.0 to 3.5 m/s. Early tumor staging, well-preserved liver function and less significant portal hypertension in our patients may account for such findings. Liver stiffness is a composite of hepatic fibrosis, inflammation, portal pressure, and other factors, while spleen stiffness might more directly reflect portal pressure only. Since portal hypertension is not the only factors associated with hepatic decompensation and mortality, liver stiffness may harbor incremental prognostic information as compared to spleen stiffness^35,39^.

There are some limitations to this study. First, the number of patients was relatively small, and the impact of LS and SS on the recurrence patterns (early or late recurrence) could not be assessed. Even though this is the largest cohort study to investigate the role of LS or SS measured by ARFI elastography in HCC patients who received RFA, further studies with a larger sample size are still needed. Second, the value of ARFI velocity might change during the follow-up period in accordance with the degree of fibrosis, which could progress as part of the natural disease course or could be affected by the anti-viral treatment^40^. Even though our data suggested both anti-viral treatment and advanced LS were significant predictors of OS, longitudinal studies with serial measurements of ARFI velocity for LS should be performed to address this question. Third, stiffness of responding tumor was recently reported to provide a useful tool for early prediction of HCC response to local ablative therapy^41^. However, this information was limited in our study. Fourth, our study only enrolled patients for whom ARFI velocity was measurable at the time of RFA, which might have led to selection bias. Moreover, our report cannot be extended to patients who have experienced other anti‐HCC treatments, and further studies are required. Fifth, the predominant underlying liver disease in our HCC cohort was chronic viral hepatitis, which is different from Western populations. It is still undetermined whether ARFI velocity could predict the risks in HCC patients arising from steatohepatitis or alcoholic liver disease. Considering less influence by steatosis^42,43^, ARFI could still be used to determine LS in patients with hepatic steatosis and might have a role in predicting HCC risks.

In conclusion, LS measured by ARFI elastography could predict both HCC recurrence and OS in patients who underwent RFA.

## Methods

### Patients

This study prospectively enrolled 173 patients who had treatment-naive HCC and underwent RFA as a first treatment at Taipei Veterans General Hospital from January 2013 to December 2017. The diagnosis and staging of HCC were performed according to the guidelines of the American Association for the Study of Liver Disease^44^. The indications of RFA were as follows: (A) solitary tumor with size < 5 cm or 2–3 tumors all with sizes < 3 cm; (B) an absence of extra-hepatic metastasis or major vascular invasion; (C) grade A CTP classification of liver functional reserve; (D) no ascites; (E) platelet count > 50,000/mm3; and (F) no other major comorbidities that might complicate the RFA procedure (such as infections, arrhythmias, acute myocardial infarction, uncontrolled congestive heart failure, chronic obstructive pulmonary disease with acute exacerbation, or recent stroke)^23^.

The study was conducted in accordance with the ethical guidelines of the 1975 Declaration of Helsinki and current ethical guidelines. It was also approved by the Institutional Review Board, Taipei Veterans General Hospital. Informed consent was obtained from all patients before the entered the study.

### RFA and follow‐up

The RFA device, procedure, and follow-up have been described previously^23,32^. Briefly, RFA was performed by experienced hepatologists or interventional radiologists with the confirmation of complete ablation. Regular follow-up with clinical assessment were performed one month after RFA and every 3-6 months later to monitor HCC recurrence. The procedures involved physical examination, laboratory exams including serum AFP levels, and contrast-enhanced image studies by computed tomography scans or magnetic resonance imaging^44,45^. All of the patients were followed until the end of 2018. The primary endpoint of this study was HCC recurrence, and the second endpoint was OS.

### Biochemical and serologic markers

Serum biochemistries were measured using a Roche/Hitachi Modular Analytics System (Roche Diagnostics GmbH, Mannheim, Germany). The serum level of AFP was tested using a radio-immunoassay kit (Serono Diagnostic SA, Coinsin/VD, Switzerland). Serum levels of hepatitis B surface antigen (HBsAg) and hepatitis C virus antibody were tested by radio-immunoassay (Abbott Laboratories, North Chicago, IL) and second-generation enzyme immunoassay (Abbott). Previously described methods were used to calculate the ratio (AAR) of AST to ALT, APRI, and ALBI grade^46–48^.

### Acoustic radiation force impulse elastography measurements

ARFI elastography was performed in fasting status of the patient^49^ to assess LS and SS on the same day as RFA with targeted selection of the non-tumor part by an experienced technician who was blinded to the clinical information of the patients. The detailed technique of ARFI elastography has been reported in previous studies, and the results are expressed in meters per second (m/s)^11^. A result is considered to be reliable when 10 validated measurements are within a ratio of IQR to the median value (IQR/M) of less than 0.3 with a success rate of more than 60%^50^.

### Statistical analysis

Data are shown as the median (IQR) or n (%) values as appropriate. Variables were compared using the chi‐squared test or Fisher’s exact test for categorical values and a student’s t‐test or the Mann–Whitney test for continuous values. The correlation between LS and SS was investigated using the Spearman correlation test. The optimal cutoff values of LS and SS were assessed using the area under receiver operating characteristic curves (AUROC). The value with the highest Youden’s Index (sensitivity + specificity − 1) was considered as the optimal cut-off. Competing risk Kaplan–Meier was applied to estimate RFS^51,52^; and OS was estimated by Kaplan–Meier method and compared using Cox’s proportional hazards model. 1:1 propensity score-matched analysis was also performed by greedy 8 to 1 digit match algorithm without replacement to reduce confounders, including tumor numbers, serum levels of AFP and total bilirubin, and viral hepatitis status. The factors associated with HCC recurrence and OS were identified by applying a multivariate forward stepwise logistic regression model using significant variables in the univariate analysis. We also performed two models in the multivariate analysis, dimensional ARFI velocity value in model I and dichotomous ARFI value in the model II, respectively. A two-tailed value of *P* < 0.05 was considered statistically significant. All statistical analyses were performed using IBM SPSS Statistics for Windows, version 21.0 (IBM Corp., Armonk, NY, USA).

## Supplementary information

Supplementary Table.

## Data Availability

The datasets generated during and analyzed during the current study are available from the corresponding author on reasonable request.
