# Supplementary material for: Liver stiffness measured by acoustic radiation force impulse elastography predicted prognoses of hepatocellular carcinoma after radiofrequency ablation
Source: Sci Rep. 2020 Feb 6;10:2006. doi: 10.1038/s41598-020-58988-3 (PMC7005159; doi:10.1038/s41598-020-58988-3)
Supplement: Supplementary file 1 — Supplementary Table. [file 41598_2020_58988_MOESM1_ESM.docx]

**SUPPLEMENTARY INFORMATION**

**Title of manuscript: Liver stiffness measured by acoustic radiation force impulse elastography predicted prognoses of** **hepatocellular carcinoma after radiofrequency ablation**

**Author list:**

Pei-Chang Lee, Yi-You Chiou, Nai-Chi Chiu, Ping-Hsien Chen, Chien-An Liu,

Wei-Yu Kao, Teh-Ia Huo, Yi-Hsiang Huang, Ming-Chih Hou, Han-Chieh Lin,

Jaw-Ching Wu, Chien-Wei Su

**Supplementary Table
Analysis of factors associated with OS and RFS in patients with
clinically significant portal hypertension^#^**

|  |  | Univariate | | |
| --- | --- | --- | --- | --- |
|  |  | HR | 95% CI | *p* value |
| Predicting OS |  |  |  |  |
| ARFI, m/s (spleen) |  | 1.023 | 0.492 – 2.126 | 0.951 |
|  | >3.0 vs. ≦3.0 | 1.116 | 0.469 – 2.657 | 0.804 |
|  |  |  | Univariate |  |
|  |  | SHR | 95% CI | *p* value |
| Predicting RFS |  |  |  |  |
| ARFI, m/s (spleen) |  | 1.177 | 0.643 – 2.153 | 0.598 |
|  | >3.0 vs. ≦3.0 | 1.824 | 0.798 – 4.169 | 0.154 |

^#^Clinically significant portal hypertension is defined as hepatic venous pressure
gradient ≧ 10mmHg or with surrogates of portal hypertension, including
esophageal varices, or splenomegaly with platelet count < 100,000/mm^3^.

Abbreviations: OS, overall survival; RFS, recurrence-free survival; ARFI, acoustic radiation force impulse; HR, hazard ratio; CI, confidence interval; SHR, subdistribution hazard ratio.
